# Supplementary figures and images for: Pervasive promoter hypermethylation of silenced TERT alleles in human cancers
Source: Cell Oncol (Dordr). 2020 May 28;43(5):847–61. doi: 10.1007/s13402-020-00531-7 (PMC7581602; doi:10.1007/s13402-020-00531-7)

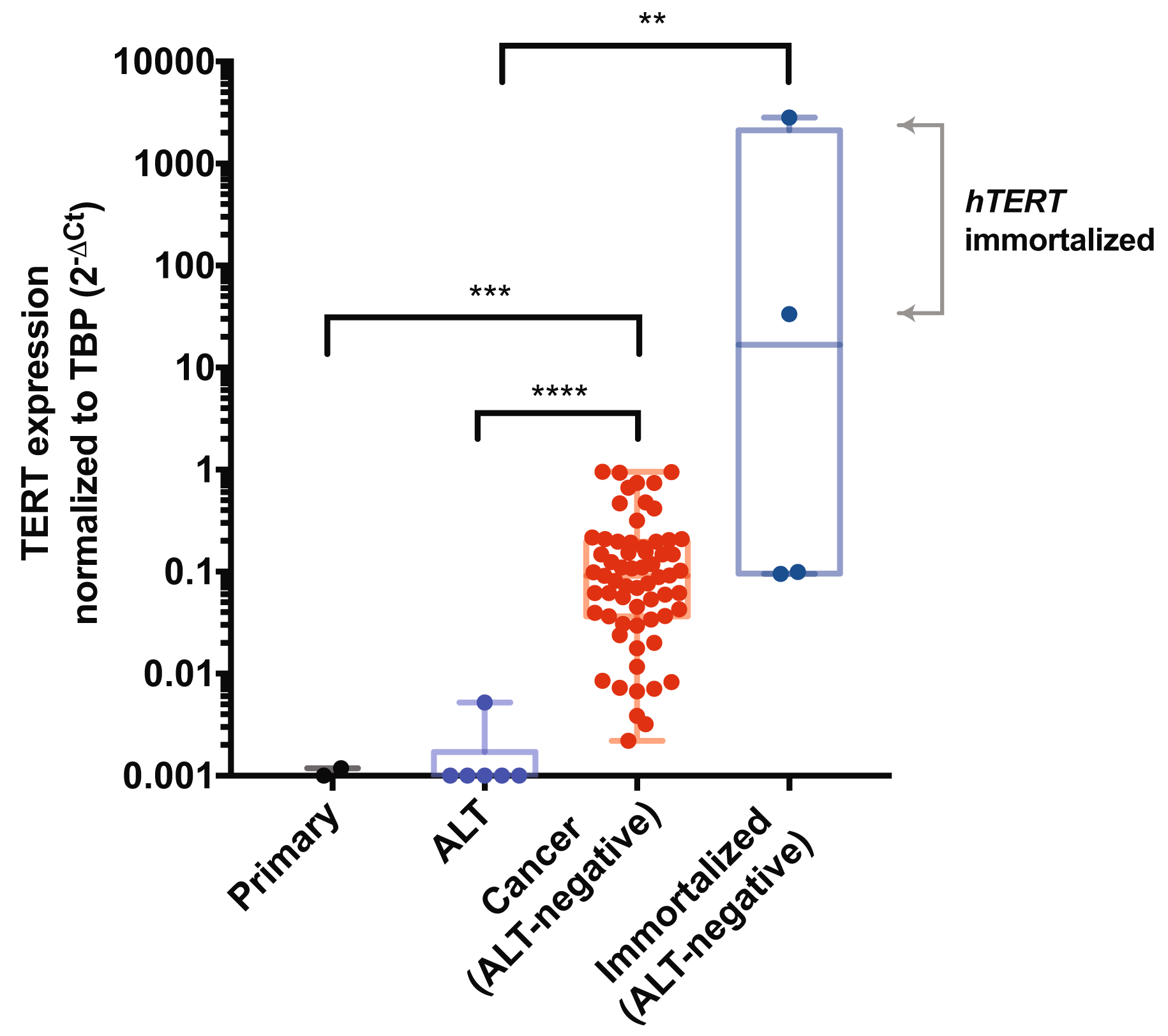

Supplement: Supplementary file 1 — (PNG 114 kb) [file 13402_2020_531_Fig6_ESM.png]

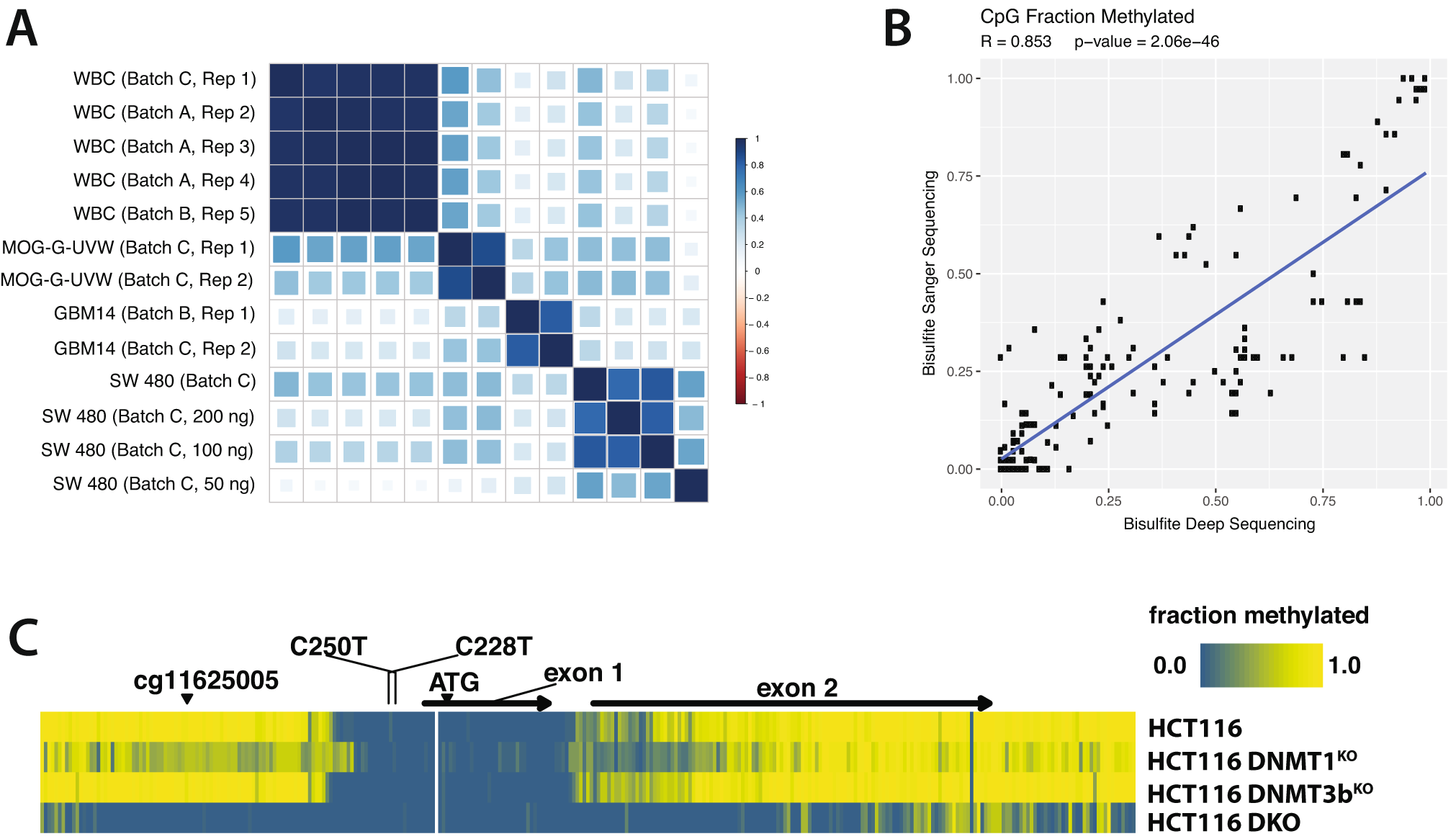

Supplement: Supplementary file 3 — (PNG 158 kb) [file 13402_2020_531_Fig7_ESM.png]

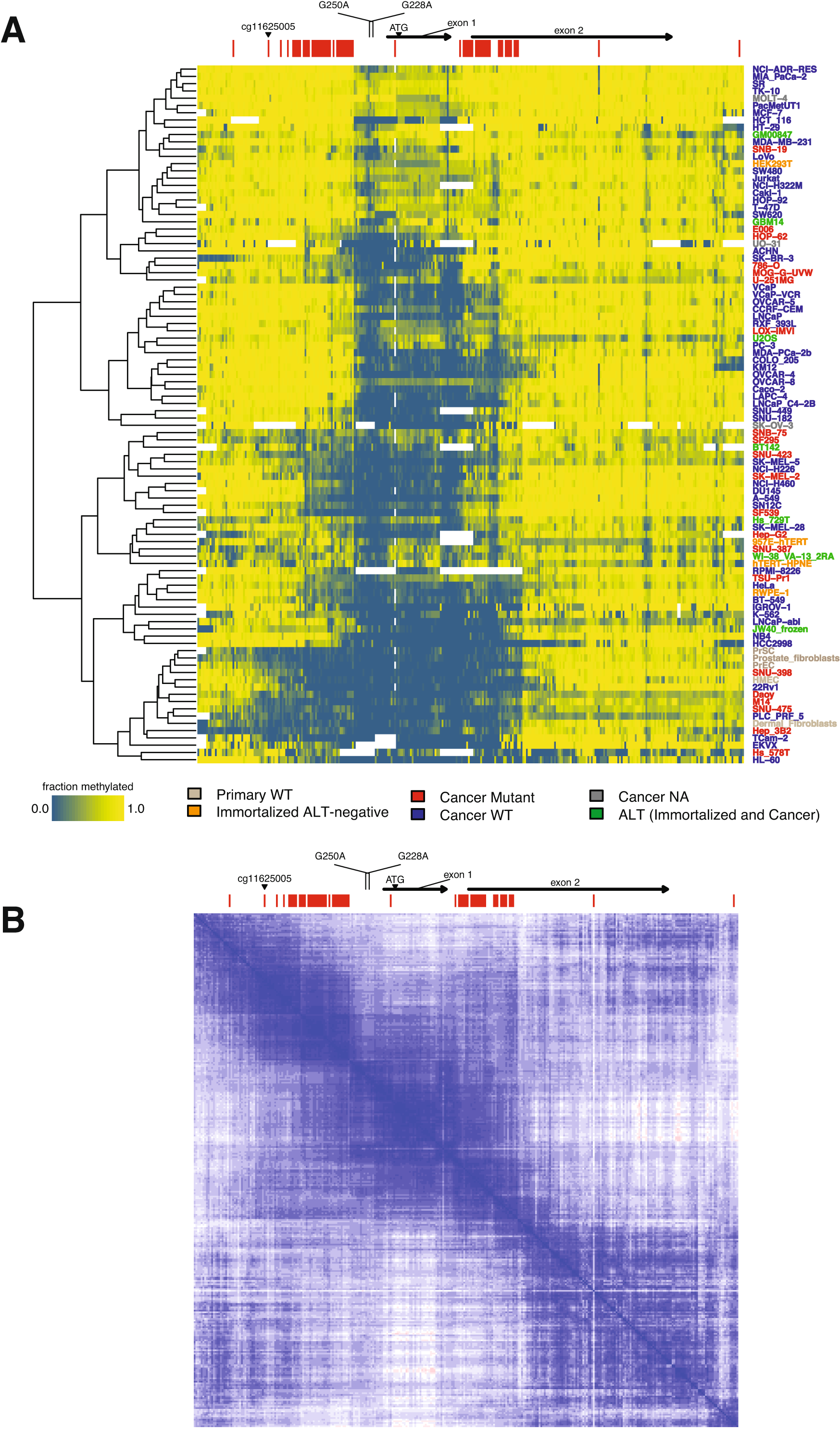

Supplement: Supplementary file 5 — (PNG 686 kb) [file 13402_2020_531_Fig8_ESM.png]

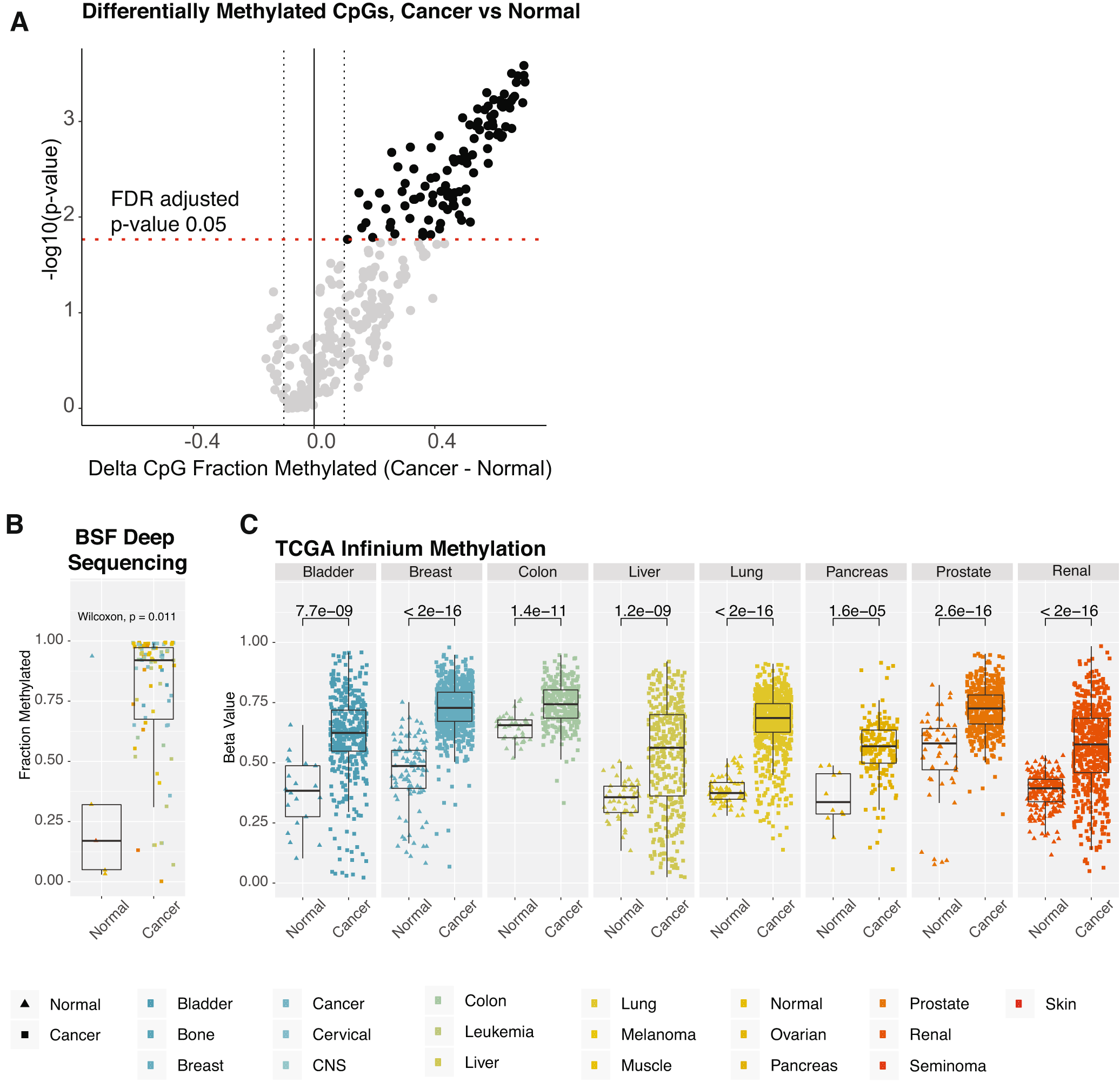

Supplement: Supplementary file 7 — (PNG 287 kb) [file 13402_2020_531_Fig9_ESM.png]

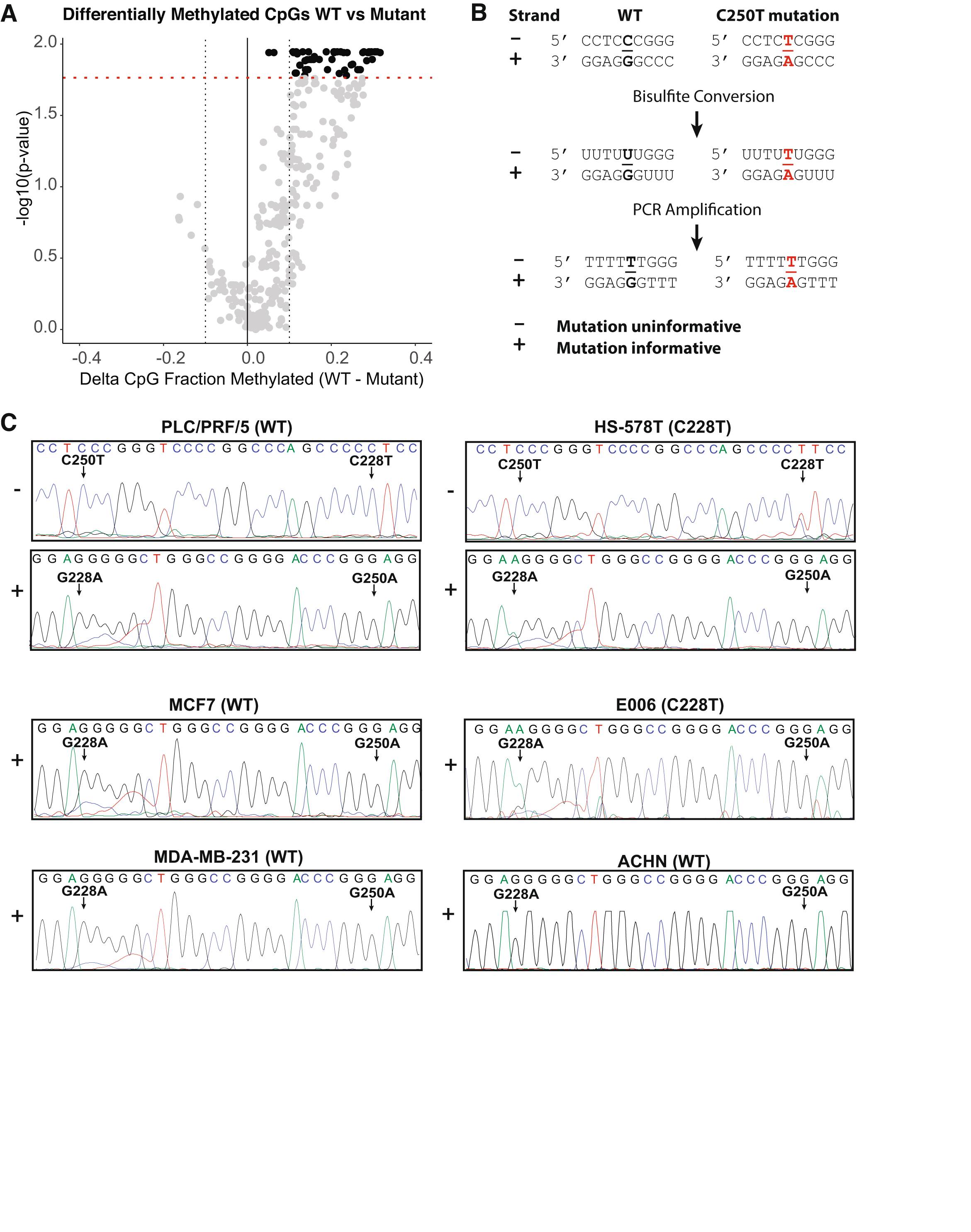

Supplement: Supplementary file 9 — (PNG 490 kb) [file 13402_2020_531_Fig10_ESM.png]

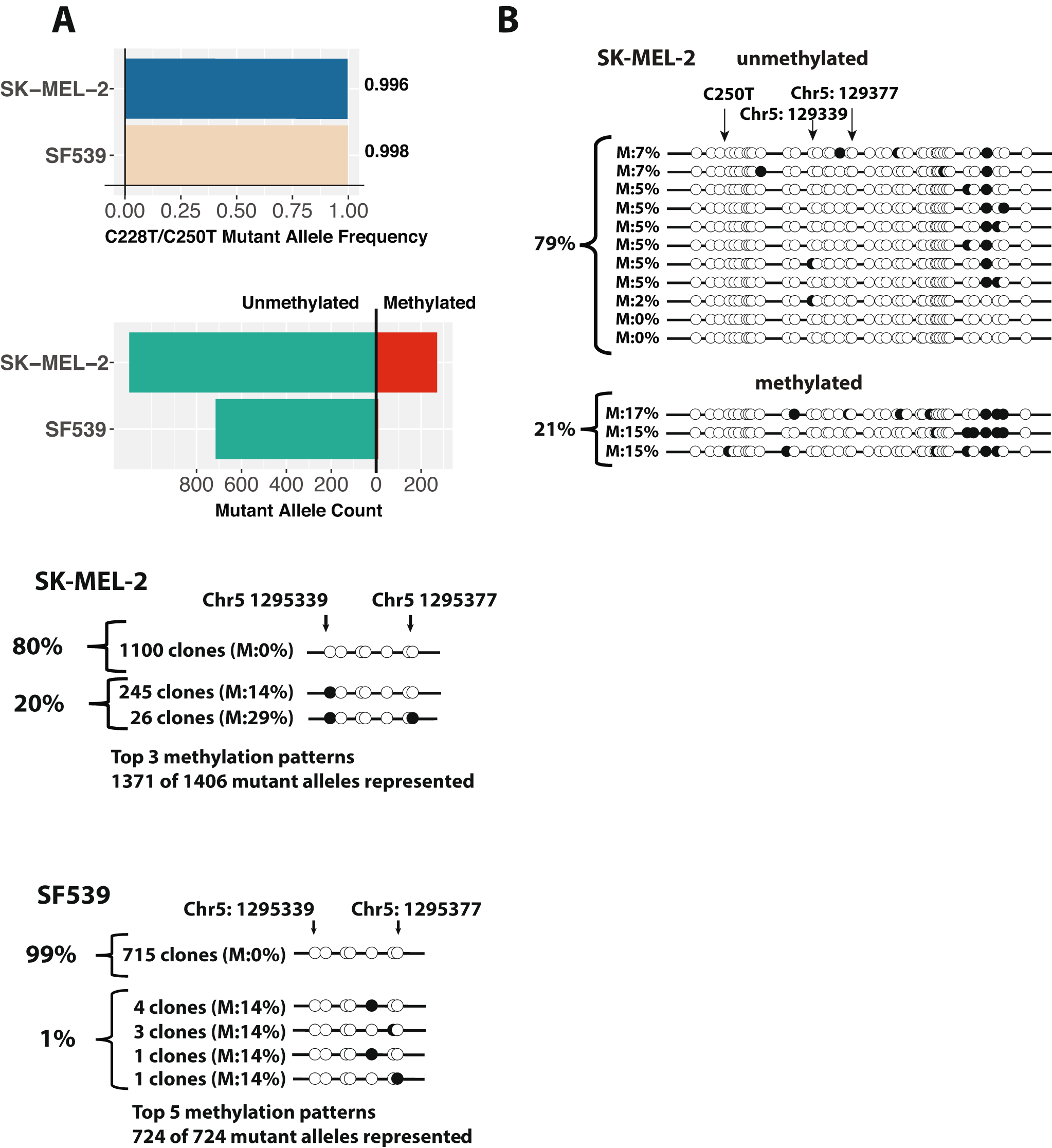

Supplement: Supplementary file 11 — (PNG 293 kb) [file 13402_2020_531_Fig11_ESM.png]

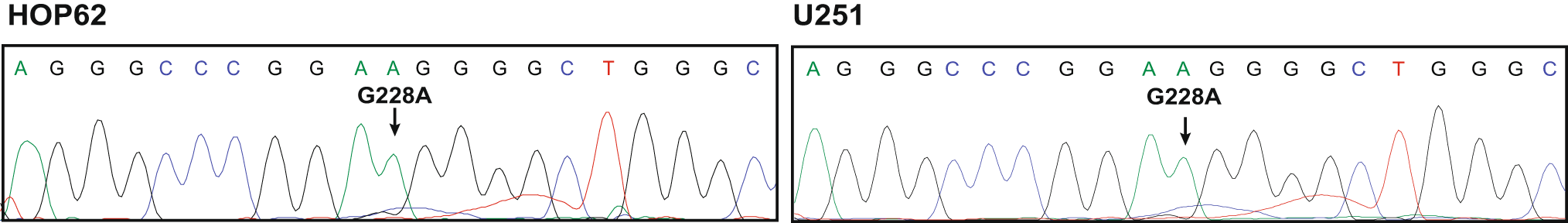

Supplement: Supplementary file 13 — (PNG 72 kb) [file 13402_2020_531_Fig12_ESM.png]

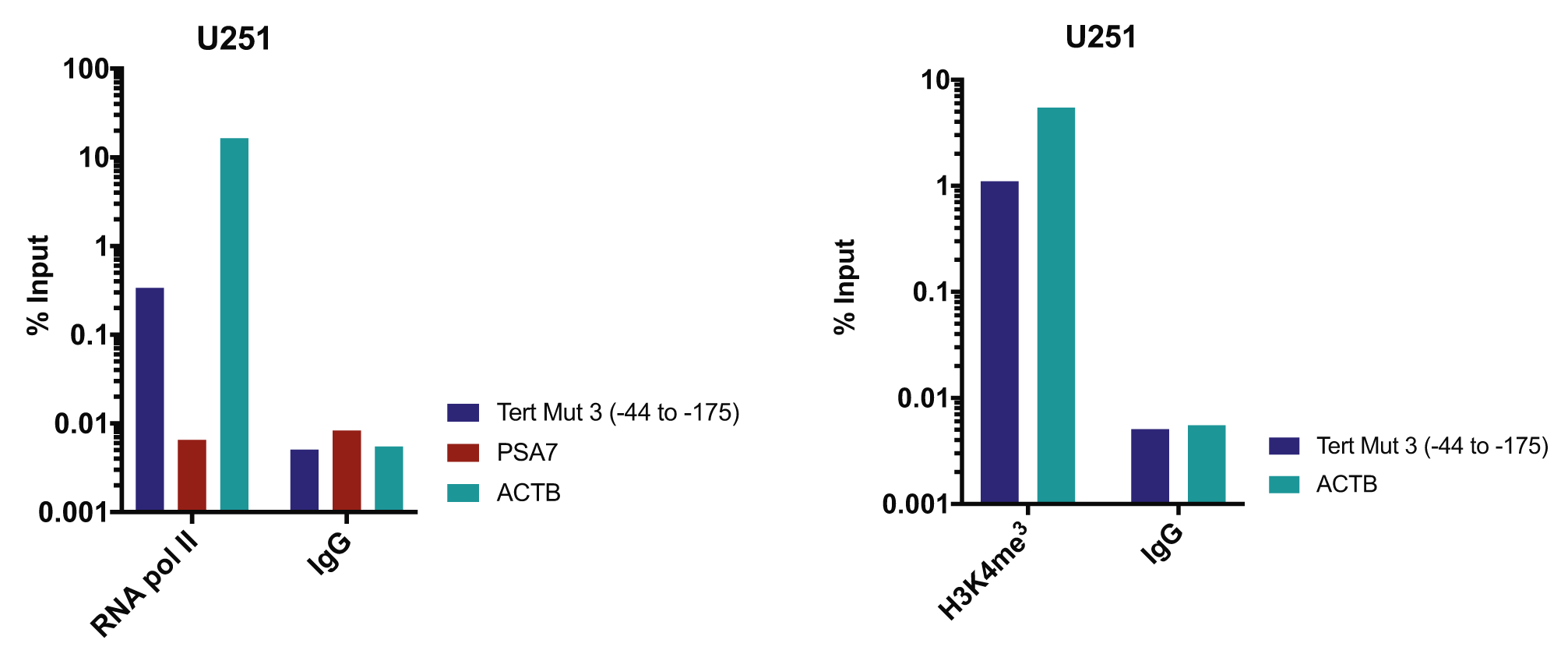

Supplement: Supplementary file 15 — (PNG 46 kb) [file 13402_2020_531_Fig13_ESM.png]

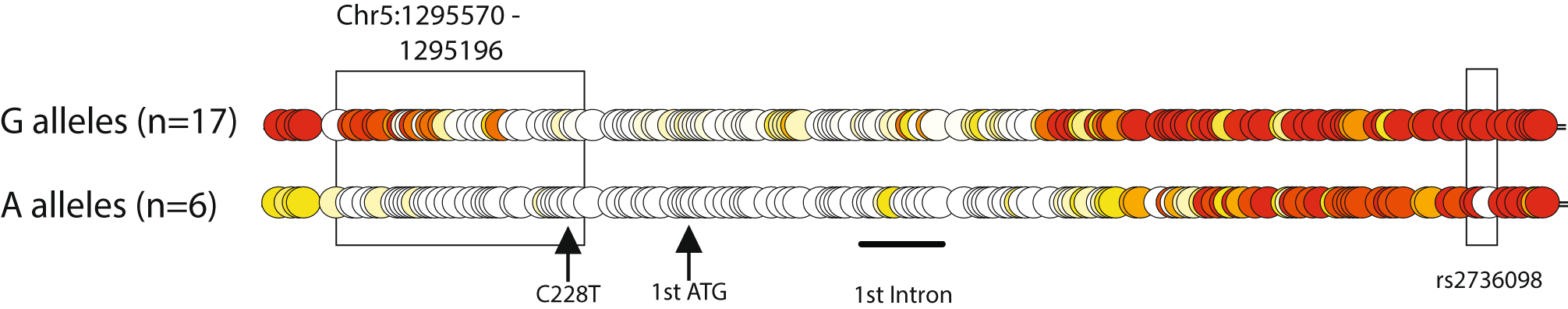

Supplement: Supplementary file 17 — (PNG 116 kb) [file 13402_2020_531_Fig14_ESM.png]

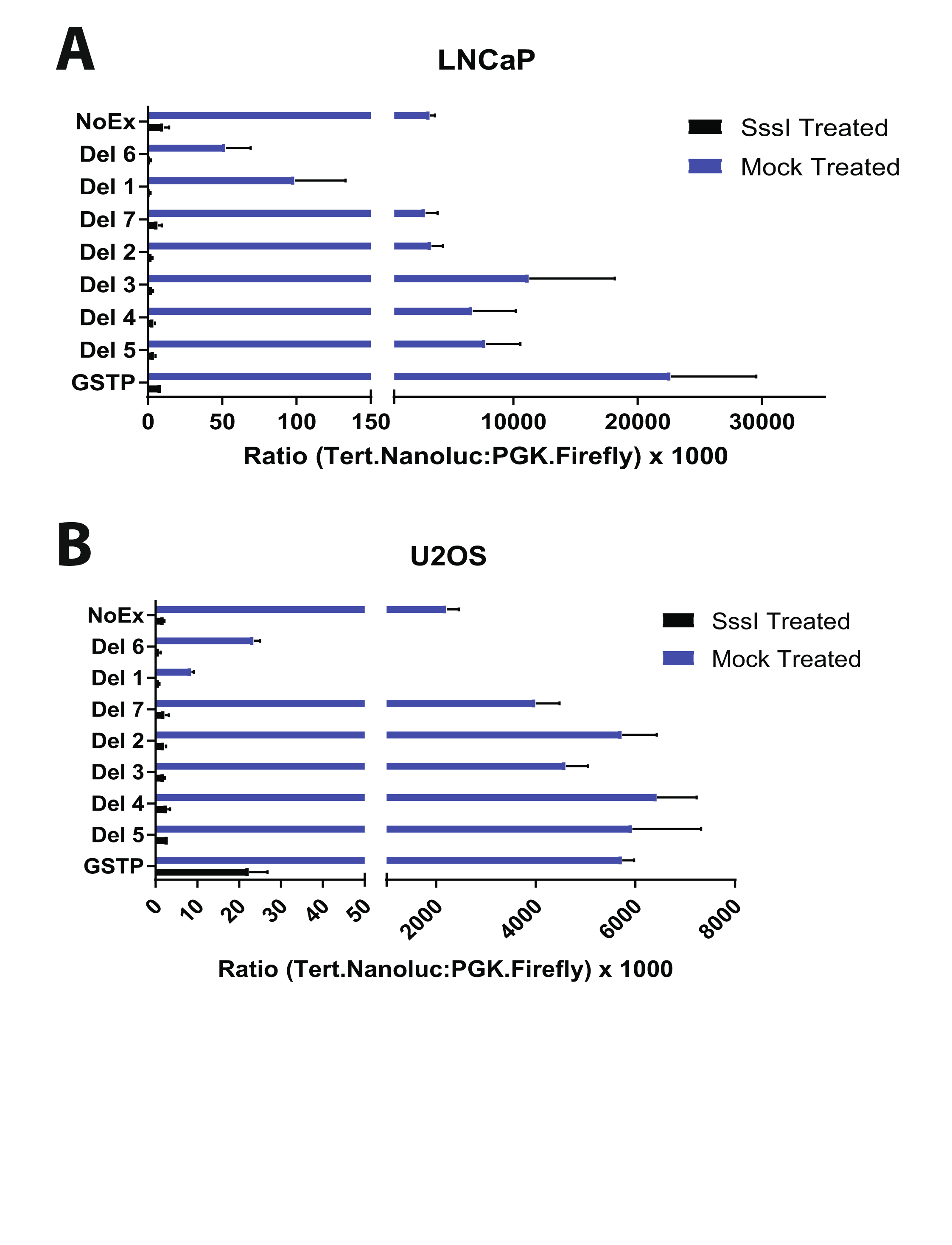

Supplement: Supplementary file 19 — (PNG 137 kb) [file 13402_2020_531_Fig15_ESM.png]
