# Supplementary material for: Pervasive promoter hypermethylation of silenced TERT alleles in human cancers
Source: Cell Oncol (Dordr). 2020 May 28;43(5):847–61. doi: 10.1007/s13402-020-00531-7 (PMC7581602; doi:10.1007/s13402-020-00531-7)
Supplement: Supplementary file 21 — (DOCX 22 kb) [file 13402_2020_531_MOESM11_ESM.docx]

**Table S1. Cell line characteristics**

| Cell Line | Tissue  Type | Cell  Type ^a^ | TERT  Promoter ^b^ | ALT  Status ^c^ | STR  Status ^d^ |  | Cell Line | Tissue  Type | Cell  Type ^a^ | TERT  Promoter ^b^ | ALT  Status ^c^ | STR  Status ^d^ |
| --- | --- | --- | --- | --- | --- | --- | --- | --- | --- | --- | --- | --- |
| Dermal_Fibroblasts | Skin | Primary | WT | Negative | Pass |  | 22Rv1 | Prostate | Cancer | WT | Negative | Pass |
| HMEC | Breast | Primary | WT | Negative | Pass |  | A-549 | Lung | Cancer | WT | Negative | Pass |
| PrEC | Prostate | Primary | WT | Negative | Pass |  | ACHN | Renal | Cancer | WT | Negative | Pass |
| Prostate_fibroblasts | Prostate | Primary | WT | Negative | Pass |  | BT-549 | Breast | Cancer | WT | Negative | Pass |
| PrSC | Prostate | Primary | WT | Negative | Pass |  | CCRF-CEM | Leukemia | Cancer | WT | Negative | Pass |
| RWPE-1 | Prostate | Imm_HPV | WT | Negative | Pass |  | COLO_205 | Colon | Cancer | WT | Negative | Pass |
| GM00847 | Fibroblast | Imm_SV40 | WT | ALT | Pass |  | DU145 | Prostate | Cancer | WT | Negative | Pass |
| WI-38_VA-13_2RA | Lung | Imm_SV40 | WT | ALT | Pass |  | EKVX | Lung | Cancer | WT | Negative | Pass |
| HEK293T | Renal | Imm_SV40 | WT | Negative | Pass |  | HCC2998 | Colon | Cancer | WT | Negative | Pass |
| 957E-hTERT | Prostate | Imm_TERT | WT | Negative | Pass |  | HCT_116 | Colon | Cancer | WT | Negative | Pass |
| hTERT-HPNE | Pancreas | Imm_TERT | WT | Negative | Pass |  | HL-60 | Leukemia | Cancer | WT | Negative | Pass |
| TSU-Pr1 | Bladder | Cancer | Mut | NA | NA |  | HOP-92 | Lung | Cancer | WT | Negative | Pass |
| 786-O | Renal | Cancer | Mut | Negative | Pass |  | HT-29 | Colon | Cancer | WT | Negative | Pass |
| Daoy | CNS | Cancer | Mut | Negative | Pass |  | IGROV-1 | Ovarian | Cancer | WT | Negative | Pass |
| E006 | Prostate | Cancer | Mut | Negative | Pass |  | K-562 | Leukemia | Cancer | WT | Negative | Pass |
| Hep_3B2 | Liver | Cancer | Mut | Negative | Pass |  | KM12 | Colon | Cancer | WT | Negative | Pass |
| Hep-G2 | Liver | Cancer | Mut | Negative | Pass |  | LAPC-4 | Prostate | Cancer | WT | Negative | Pass |
| HOP-62 | Lung | Cancer | Mut | Negative | Pass |  | LNCaP | Prostate | Cancer | WT | Negative | Pass |
| Hs_578T | Breast | Cancer | Mut | Negative | Pass |  | LNCaP_C4-2B | Prostate | Cancer | WT | Negative | Pass |
| LOX-IMVI | Melanoma | Cancer | Mut | Negative | Pass |  | LNCaP-abl | Prostate | Cancer | WT | Negative | Pass |
| M14 | Melanoma | Cancer | Mut | Negative | Pass |  | MCF-7 | Breast | Cancer | WT | Negative | Pass |
| MOG-G-UVW | CNS | Cancer | Mut | Negative | Pass |  | MDA-MB-231 | Breast | Cancer | WT | Negative | Pass |
| SF295 | CNS | Cancer | Mut | Negative | Pass |  | MDA-PCa-2b | Prostate | Cancer | WT | Negative | Pass |
| SF539 | CNS | Cancer | Mut | Negative | Pass |  | NCI-ADR-RES | Ovarian | Cancer | WT | Negative | Pass |
| SK-MEL-2 | Melanoma | Cancer | Mut | Negative | Pass |  | NCI-H226 | Lung | Cancer | WT | Negative | Pass |
| SNB-19 | CNS | Cancer | Mut | Negative | Pass |  | NCI-H322M | Lung | Cancer | WT | Negative | Pass |
| SNB-75 | CNS | Cancer | Mut | Negative | Pass |  | NCI-H460 | Lung | Cancer | WT | Negative | Pass |
| SNU-387 | Liver | Cancer | Mut | Negative | Pass |  | OVCAR-4 | Ovarian | Cancer | WT | Negative | Pass |
| SNU-398 | Liver | Cancer | Mut | Negative | Pass |  | OVCAR-5 | Ovarian | Cancer | WT | Negative | Pass |
| SNU-423 | Liver | Cancer | Mut | Negative | Pass |  | OVCAR-8 | Ovarian | Cancer | WT | Negative | Pass |
| SNU-475 | Liver | Cancer | Mut | Negative | Pass |  | PacMetUT1 | Prostate | Cancer | WT | Negative | Pass |
| U-251MG | CNS | Cancer | Mut | Negative | Pass |  | PC-3 | Prostate | Cancer | WT | Negative | Pass |
| MOLT-4 | Leukemia | Cancer | NA | Negative | Pass |  | PLC_PRF_5 | Liver | Cancer | WT | Negative | Pass |
| SK-OV-3 | Ovarian | Cancer | NA | Negative | Pass |  | RPMI-8226 | Leukemia | Cancer | WT | Negative | Pass |
| UO-31 | Renal | Cancer | NA | Negative | Pass |  | RXF_393L | Renal | Cancer | WT | Negative | Pass |
| BT142 | CNS | Cancer | WT | ALT | Pass |  | SK-MEL-28 | Melanoma | Cancer | WT | Negative | Pass |
| GBM14 | CNS | Cancer | WT | ALT | Pass |  | SK-MEL-5 | Melanoma | Cancer | WT | Negative | Pass |
| Hs_729T | Muscle | Cancer | WT | ALT | Pass |  | SN12C | Renal | Cancer | WT | Negative | Pass |
| JW40_frozen | Prostate | Cancer | WT | ALT | Pass |  | SNU-182 | Liver | Cancer | WT | Negative | Pass |
| U2OS | Bone | Cancer | WT | ALT | Pass |  | SNU-449 | Liver | Cancer | WT | Negative | Pass |
| Caco-2 | Colon | Cancer | WT | NA | NA |  | SR | Leukemia | Cancer | WT | Negative | Pass |
| Caki-1 | Renal | Cancer | WT | NA | NA |  | SW620 | Colon | Cancer | WT | Negative | Pass |
| HeLa | Cervical | Cancer | WT | NA | Pass |  | T-47D | Breast | Cancer | WT | Negative | Pass |
| Jurkat | Leukemia | Cancer | WT | NA | NA |  | TK-10 | Renal | Cancer | WT | Negative | Pass |
| LoVo | Colon | Cancer | WT | NA | NA |  | VCaP | Prostate | Cancer | WT | Negative | Pass |
| MIA_PaCa-2 | Pancreas | Cancer | WT | NA | NA |  | VCaP-VCR | Prostate | Cancer | WT | Negative | Pass |
| NB4 | Leukemia | Cancer | WT | NA | NA |  |  |  |  |  |  |  |
| SK-BR-3 | Breast | Cancer | WT | NA | Pass |  |  |  |  |  |  |  |
| SW480 | Colon | Cancer | WT | NA | Pass |  |  |  |  |  |  |  |
| TCam-2 | Seminoma | Cancer | WT | NA | Pass |  |  |  |  |  |  |  |

^a^ Cells types include primary, immortalized or cancer. Imm_HPV indicates an HPV immortalized cell line. Imm_SV40 indicates an SV40 immortalized cell line. Imm_TERT indicates that the cell line was immortalized b overexpressing the catalytic subunit of telomerase, *TERT*.

^b^ Cell lines with TERT promoter mutations are indicated as “Mut” for mutant or “WT” for wild type.

^c^ Cell lines that employ the telomerase-independent telomere maintenance mechanism, Alternative lengthening of Telomeres (ALT), are indicated as “ALT”.

^d^ Authentication of human cell lines using STR DNA profiling.
