# Supplementary material for: Pervasive promoter hypermethylation of silenced TERT alleles in human cancers
Source: Cell Oncol (Dordr). 2020 May 28;43(5):847–61. doi: 10.1007/s13402-020-00531-7 (PMC7581602; doi:10.1007/s13402-020-00531-7)
Supplement: Supplementary file 22 — (DOCX 18 kb) [file 13402_2020_531_MOESM12_ESM.docx]

**Table S2. Primers used to prepare bisulfite sequencing amplicon libraries**

| Amplicon | Strand | Amplicon Size (bp) | Coordinates (Hg19/GRCh37) | Forward Primer (5'-3') | Reverse Primer (5'-3') |
| --- | --- | --- | --- | --- | --- |
| Tert_1+ | plus | 272 | 1295525 1295796 | GGTTTAGGTTGTGGGGTAATT | AAAAAATATTACAAAAAAACACTCC |
| Tert 14 | plus | 248 | 1291967 1292214 | GATTAGAGAATTTAAATTTTTTTTAA | CATAAAATTAACACTCCTAACAC |
| Tert 15 | plus | 162 | 1292313 1292474 | GGGAGTTTGTGGGGAGGGGGTGAA | TCAAAAAATAATACTACTAAACCCTAC |
| Tert 16 | plus | 144 | 1292603 1292746 | TTATTTTGTTTTGGTATAATTAA | CTAAAACAAAAAAATCACTTAAACCC |
| Tert 17 | plus | 253 | 1292879 1293131 | TAGTTTTTTAAAGTGTTGGGA | ACAATAAAAAAAAATATCTAAAAACAC |
| Tert 18 | plus | 254 | 1293101 1293354 | GTTTGTGTTTTTAGATATTTTTTTTTA | CAAAACCCTAATCCTCCTATCTCCAT |
| Tert 19 | plus | 262 | 1293381 1293642 | GTATTTAGTTTTGGGGTTTGGGT | CCAACACAACAACCCCTAACAAAT |
| Tert_2- | plus | 297 | 1293895 1294191 | TTGGAATTTAGAAAGATGGTTTTTA | CTATATAATATCACCTACCAAACCC |
| Tert 20 | plus | 213 | 1293618 1293830 | TATTTGTTAGGGGTTGTTGTGTTG | CCCCTATTTCTAAAACTACTTAAAAAC |
| Tert 21 | plus | 214 | 1293808 1294021 | TTAAGTAGTTTTAGAAATAGGGG | AAACCAAACACTTCCTCTACTCCTC |
| Tert 22 | plus | 202 | 1293996 1294197 | TGAGGAGTAGAGGAAGTGTTTGGTT | TAATTTCTATATAATATCACCTACC |
| Tert 23 | plus | 197 | 1294173 1294369 | GGTAGGTGATATTATATAGAAA | AAATCCCCCTAAACCTACCAACCC |
| Tert 25 | plus | 247 | 1294538 1294784 | GTGTTAGTAGGTGAATTAGTA | CAAATATCCTACCTAAAAAAACTAATAAC |
| Tert 26 | plus | 255 | 1294754 1295008 | GGGTTATTAGTTTTTTTAGGTAGGATA | CCCCAAAACTAACRACTAATACAAC |
| Tert 27 | plus | 201 | 1294934 1295134 | GTATATTAGGTATTGGGTTATTA | TAAAAAACCCTAACCCCRACCACCC |
| Tert 28 | plus | 228 | 1294996 1295223 | GTTAGTTTTGGGGTTTTAGG | AACCCTCCCAACCCCTCCCCTTCCTT |
| Tert 29 | plus | 207 | 1295197 1295403 | AAAGGAAGGGGAGGGGTTGGGAGGG | AAACCAAACCRAACTCCCAATAAAT |
| Tert_2b | minus | 218 | 1293882 1294162 | AAGAAGTTATTTTTTTGGAGGGTG | TAACATCCAAAACCTAAAACCCA |
| Tert 30 | plus | 257 | 1295314 1295570 | GGGTGTTYGGGTTTTTAGTTTTT | CCTCCACATCATAACCCCTCCCTC |
| Tert 31 | plus | 242 | 1295555 1295796 | GGTTATGATGTGGAGGTTTTGGGAATAGG | AAAAAATATTACAAAAAAACACTCC |
| Tert 32 | plus | 229 | 1295716 1295944 | GGTTGGGGATGAATTYGAGGA | CCTAACTCCATTTCCCACCCTTTCT |
| Tert 33 | plus | 231 | 1295919 1296149 | GAGAAAGGGTGGGAAATGGAGTTAG | CTTCTACTACTAAACTAAAAATC |
| Tert 34 | plus | 209 | 1296127 1296335 | GATTTTTAGTTTAGTAGTAGAAG | TTTATTAACATTTCAATATTTACC |
| Tert 35 | plus | 241 | 1296283 1296523 | GGTTTTGTAGGGATGTTGTAGTTGAGG | CTTAAAAATCACTAAAAAAATTTCT |
| Tert 37 | minus | 257 | 1296158 1296414 | GTTTAAATGTTAGTTTTATAAATAAAG | CCACTAATCCCCTCCAAACCT |
| Tert 38 | minus | 215 | 1296018 1296232 | GGAGTTTGGATTTTTGGGAAGTTTTTAG | CTAACAAATAAAACCAACATCTAATCAC |
| Tert 39 | minus | 213 | 1295826 1296038 | GATGTTGGTTTTATTTGTTAGATAGAG | TCATTTCTCTTTACAAATTCTCA |
| Tert 40 | minus | 231 | 1295621 1295851 | GTTTGAGAATTTGTAAAGAGAAATG | AAACCCAAAACTACCTCCAAATCC |
| Tert 41 | minus | 172 | 1295473 1295644 | GGATTTGGAGGTAGTTTTGGGTTT | CTCCCAAAATACAAAAACRCCAAC |
| Tert 42 | minus | 209 | 1295378 1295586 | GTATTTGTTTTTAGGGTTTTTATATTATG | AATCCACTAAAAACCCRACCTAACCC |
| Tert 43 | minus | 205 | 1295292 1295496 | GTTGGYGTTTTTGTATTTTGGGAG | CTAAAAAATAAAAAAACAAAACRAATACC |
| Tert 44 | minus | 235 | 1295109 1295343 | GTGGYGGAGGGATTGGGGATT | AAAATAACCRAAACCAAAACTTCCC |
| Tert 45 | minus | 255 | 1294985 1295239 | GGTTTAGTTTTTTTYGGGTTTTTTTAG | CTACACCAACCRCCAACCCTAAAACCCC |
| Tert 47 | minus | 247 | 1294539 1294785 | GTAGGTGTTTTGTTTGAAGGAGTTGGTGG | TACCAACAAATAAACCAACAC |
| Tert 48 | minus | 211 | 1294349 1294559 | GTGTTGGTTTATTTGTTGGTA | CTAACAAACCCAAAAAAACCCC |
| Tert 49 | minus | 232 | 1294139 1294370 | GGGGTTTTTTTGGGTTTGTTAG | CACCCTCCAAAAAAATAACTTCTT |
| Tert 52 | minus | 216 | 1293616 1293831 | GTTTTTGTTTTTGGAGTTGTTTGGGAA | TACACCTACCAAAAACTACTATACT |
| Tert 53 | minus | 231 | 1293413 1293643 | GTTAGTATAGTAGTTTTTGGTAGGTGTA | CCACCACCTCCTCACCTAAACTCCT |
| Tert 55 | minus | 221 | 1293156 1293376 | GGGGTTTAGAAAAGGGGGTAGGTAGAG | CCCAACCTCCTCTATTCACTACTCTAACC |
| Tough_tert_4 | minus | 183 | 1294348 1294530 | GTTTTTTGTGTTGGTGGTTTTTA | ACTAACAAACCCAAAAAAACCCC |
